# The Shifting Cultivation Juggernaut: An Attribution Problem

**DOI:** 10.1002/gch2.202200051

**Published:** 2022-06-10

**Authors:** Arun Jyoti Nath, Demsai Reang, Gudeta W. Sileshi

**Affiliations:** ^1^ Department of Ecology and Environmental Science Assam University Silchar 788011 India; ^2^ Department of Plant Biology and Biodiversity Management Addis Ababa 3434 Ethiopia

**Keywords:** ethnic marginalization, indigenous farming system, sustainable forest management, tropical deforestation

## Abstract

Shifting cultivation entails clearing a delimited land and transforming it into arable land. Owing to its complexity, this system has been a subject of debate and intervention since the colonial‐era, and is often considered as the “tropical deforestation culprit.” Shifting cultivators are often labeled as “forest eaters” and are considered backward and primitive. Opponents of shifting cultivation often attribute the loss of forest cover to shifting cultivation, and favor intensification, claiming that commercial plantations are more productive. However, attempts to replace it have often failed due to inadequate understanding of the system and the decision‐making processes involved. On the other hand, a growing body of literature provides evidence that shifting cultivation is an ecologically and economically efficient practice. After a careful review of the literature, the authors conclude that the dichotomy of opinions is the consequence of the attribution problem. The authors also argue that the management of forest ecosystems will be challenging if policy and practice are not based on careful understanding of the power of this age‐old practice. Hence, there is a need for a careful diagnosis of this system and a rethink before claiming that the system is unsustainable and attempting to replace it with practices such as plantations.

## Graphical abstract

Shifting cultivation is practiced widely in the tropical uplands, covering roughly 280 million hectares of land. However, divergent perspectives and multiple attributions are often offered, and some of them have long shaped negative opinions. The authors conclude that shifting cultivation remains a complex and misunderstood form of agricultural practice, and it should be accepted as a rational land use system.

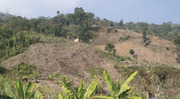

## 1 Introduction

Shifting cultivation, variously known as “slash‐and‐burn agriculture”, “swidden”, and “rotational bush fallow agriculture”, is a traditional subsistence farming long practiced by the hill farmers in the tropical highlands. It is practiced widely in the wet tropics of the world's uplands in Africa, Latin America, Oceania, and South and Southeast Asia.^[^ ^1^ ^]^ Recent estimates of its extent show that roughly 280 million hectares of land, with the largest share in Africa, followed by the Americas and Asia.^[^ ^2^ ^]^ According to Heinimann and co‐workers,^[^ ^2^ ^]^ 62% of the investigated one‐degree cells in the humid and sub‐humid tropics currently show signs of shifting cultivation, the majority being in the Americas (41%) and Africa (37%).

**Figure 1:**
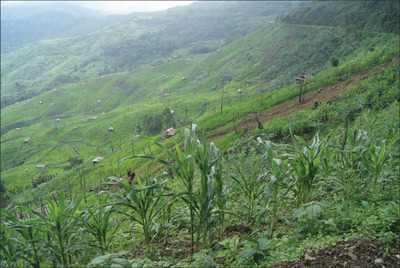
Landscape view of shifting cultivation practice in the Indian Eastern Himalayan region. Photo credit (Krishna Giri).

**Figure 2:**
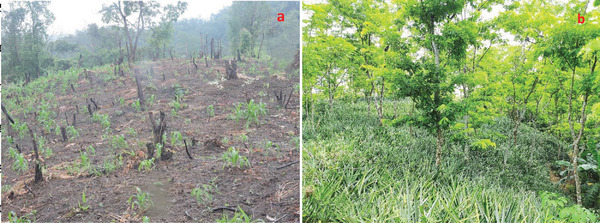
Transition from shifting cultivation (a) to traditional pineapple agroforestry system (b) practised by the Hmar tribe in the Indian Eastern Himalayas

Shifting cultivation (**Figures** **1** and **2**) involves clearing land off the vegetation and burning the biomass to produce charred material for soil fertility enrichment.^[^ ^3^ ^]^ Consequently, it has been seen as the primary source of forest degradation and deforestation^[^ ^4^, ^5^ ^]^ and viewed negatively as contributing to environmental degradation.^[^ ^6^ ^]^ There have been many attempts to eradicate it throughout much of the tropics. Nevertheless, this practice is widely known for providing subsistence to 200–300 million people across 64 developing countries.^[^ ^1^, ^7^ ^]^ For instance, 14–34 million people are dependent on shifting cultivation for their livelihoods in Southeast Asia alone.^[^ ^8^ ^]^ As such, shifting cultivation remains essential in many tropical countries, and even though it has generated a relatively large literature, aspects of the system are still poorly understood. Owing to its diversity and complexity, shifting cultivation has been a subject of debate and sometimes counterproductive interventions have been implemented. In this article, our objective is to examine the different perspectives for a better understanding of the system complexity and dynamics.

## 2 Complexity of the Practice

Shifting cultivation is not a monolithic practice but a complex and evolving indigenous resource management innovation. For instance, Conklin^[^ ^9^ ^]^ distinguished two fundamental types of shifting cultivation practices in the Philippines, namely partial and integral system. The former evolved predominantly from the economic interests of the farmers, while the latter is a system stemming from a more traditional, year‐round, largely self‐contained, community‐wide, and ritually sanctioned way of life. In the integral system, social bonds among community members are strengthened through such culturally imbibed practices. Forests have always been considered sacred to these farming communities with the faith that forests are a source of power for the deities.^[^ ^10^ ^]^ To a shifting cultivator, such a landscape has a soul–cultural existence like any other living thing.^[^ ^11^ ^]^ Although often practiced by the poorer segments of a population, this does not make it an unprofitable system.^[^ ^12^ ^]^ Besides, shifting cultivation is not a mere farming practice but a way of life for the cultivators.

Shifting cultivation is considered adaptive, sound, and appropriate given the demographic limitations. The increase in human population and resultant agricultural intensification has reduced fallow periods in many regions, rendering fallow periods insufficient for forest regeneration.^[^ ^13^ ^]^ Nevertheless, there is no evidence of system collapse, even with short fallow periods.^[^ ^12^ ^]^ A growing body of evidence also shows that when shifting cultivation is discontinued, it is often replaced by intensified land uses with higher environmental impacts.^[^ ^2^ ^]^ On the other hand, secondary forest regeneration after the cultivation is an important carbon sink^[^ ^14^ ^]^ with comparable biodiversity to mature forests.^[^ ^15^ ^]^

## 3 Divergence of Perspectives

Divergent perspectives and multiple attributions are often offered about shifting cultivation, and some of them have long shaped negative opinions. As argued by Heinimann et al.,^[^ ^2^ ^]^ shifting cultivation has been subject to recycling of statements about its merits and demerits that have not been established thorough empirical research. It has also been a subject of diametrically opposing viewpoints in the literature. On one extreme, it has been cited as the major driver of deforestation and in some cases loss of forest cover was attributed to shifting cultivation. National Governments in countries like Indonesia, Thailand, Vietnam, and Philippines often condemn shifting cultivators and the ethnic minorities that practice it for the forest loss.^[^ ^16^ ^]^ Since the agriculture practice involves periodic clearing of new forest patches for cultivation shifting cultivators are also labeled as “forest eaters.”^[^ ^16^ ^]^ At the other extreme of the continuum are accounts that valorize shifting cultivators as a form of sustainable practice, representing indigenous farmers as the original environmentalists.^[^ ^17^, ^18^ ^]^ In such accounts, this traditional adaptiveness is now under threat by modern economic and cultural encroachments.

It is increasingly being clear that the attribution of forest loss to shifting cultivators has been based on inadequate evidence. A study by Khiem and Van Der Poel^[^ ^19^ ^]^ affirms that no correlation exists between the occurrence of shifting cultivation and extent of deforestation. In fact, the majority of land referred to as “deforested” was actually found in land use representing secondary regeneration or forest fallows.^[^ ^2^, ^16^ ^]^ But, we often fail to appreciate that the “fallow” is the period dedicated for vegetation to regenerate and recuperate land but not abandonment. It is a part of the land management to be brought under cultivation and from which farmers simultaneously accumulate basic resource needs while it lies fallow. Hence, failure to appreciate this cultivation system and its associated succession vegetation can result in overestimation of the deforestation attributable to shifting cultivation, particularly in Southeast Asia. Alcorn^[^ ^20^ ^]^ pointed out that the indigenous practice is a form of “managed deforestation.” Therefore, secondary regeneration occurring during the fallow period is not necessarily deforestation.^[^ ^16^ ^]^ What is evident during shifting cultivation is the change of forest structure from a fairly homogenous stand to highly heterogeneous forest cover under secondary regeneration.^[^ ^16^ ^]^ Other changes resulting from long‐term shifting cultivation practices are increases in the degree of fragmentation.^[^ ^21^ ^]^ However, empirical studies show that biodiversity in forest fragments is comparable with that in the mature forests.^[^ ^22^ ^]^ Plants and birds diversity in some areas under shifting cultivation are known to represent 50–80% of the diversity present in the natural forests,^[^ ^15^ ^]^ although large mammals may be affected due to habitat fragmentation.^[^ ^16^ ^]^

In policy debates, shifting cultivation is sometimes framed as one of the most serious land use problems in the tropics.^[^ ^23^ ^]^ A poor understanding of the farming systems has generally driven government policies to target large scale resettlement programs which were largely unsuccessful. These programs intended to convert indigenous shifting cultivators into sedentary farmers have often turned them into farm laborers by uprooting them from their traditional land.^[^ ^16^ ^]^ For example, the colonial and post‐colonial governments in Asia have constantly tried to eradicate the practice in the name of forest conservation and development for more than a century.^[^ ^24^ ^]^ As a result, several South, Southeast Asia and African countries have imposed laws and specific policies that criminalize shifting cultivators.^[^ ^5^, ^18^, ^25^ ^]^

In some regions, for example, in many parts of Asia and Africa, shifting cultivation is linked to indigenous ways of life. In Asia, ethnic groups are comparatively poorer than the rest of the population, but endowed with rich and diverse cultures.^[^ ^26^ ^]^ These cultural differences make them distinct from the mainstream society and are the root cause of their marginalization, dispossession, discrimination and impoverishment, driven by many external forces. To mention a few—the dispossession of their traditional lands, denial of access to forests resources, and traditional livelihoods including shifting cultivation. Shifting cultivators are also conflated with other negative attributes. They are regarded as backward, a hindrance to a nation's progress, a security problem for the state, etc. Hence, even though our understanding and knowledge on land use and management among these ethnic cultivators has been fairly enriched through innumerable studies, attitudes of decision makers and resource managers have hardly changed.^[^ ^24^ ^]^ The resultant negative consequences of restrictive policies against shifting cultivation and the forceful relocation of populations is likely to accelerate loss of livelihoods and the indigenous knowledge of natural plant and animal diversity.^[^ ^27^, ^28^ ^]^ There is no doubt that shifting cultivation is declining and shall continue to decline over time, raising issues of livelihood security and resilience among people currently depending on shifting cultivation.^[^ ^2^ ^]^ Several factors determine the changing trends of this cultivation system. Many colonial era narratives blame the arrival of Europeans as the prime cause of disruption of the system. The European influence led to a much more intensive cropping which was earlier thought to have no serious effect on the soil.^[^ ^29^ ^]^ In addition, modern medicines resulted in the dramatic rise of population all over the tropics since the twentieth century.^[^ ^30^ ^]^ The consequence was continuous cropping and shortening of the fallow length.^[^ ^31^ ^]^ Increasing control of land by powerful forces^[^ ^29^ ^]^ has also led to reduced land‐holding sizes by local communities and repeated cropping. Decrease in access to land also means that sections of the cultivating population, particularly the youth are forced to seek employment in the urban centers.^[^ ^32^ ^]^

Today, tropical forest and shifting agriculture are declining, while commercial plantations are continuously expanding.^[^ ^33^, ^34^, ^35^ ^]^ This trend is particularly evident in tropical Asia.^[^ ^36^ ^]^ Many of the commercial or smallholder oil palm and rubber plantations that cover large areas of Southeast Asia today are on land that was formerly used for shifting cultivation.^[^ ^2^ ^]^ Though the land use transitions from shifting cultivation to intensified plantations increases profits, the benefit came at significant costs including the loss of indigenous knowledge and customary land management practices, socio‐economic wellbeing, and livelihood options.^[^ ^37^ ^]^ Yet, opponents of shifting cultivation often favor intensification, claiming commercial systems to be more productive with fewer environmental problems compared to the indigenous shifting agriculture.^[^ ^38^ ^]^ But, cases of the transition from shifting cultivation to more intensive land uses reveal drastic negative impacts on the ecosystems leading to permanent deforestation and biodiversity loss^[^ ^39^ ^]^ including disappearance of local varieties and breeds of plants and animals. This can undermine the resilience of local food systems and pose risks to global food security. This transition can also reduce total carbon stock.^[^ ^40^ ^]^

We contend that the term “slash‐and‐burn” conveys a negative connotation, reflecting a widespread view and stereotype that it is counterproductive. Recent systematic reviews and meta‐analysis provide evidence that shifting cultivation is an ecologically and economically efficient practice^[^ ^41^, ^42^ ^]^ despite being maligned as a practice that generates low productivity and environmental degradation. The strength of this traditional land use lies in the diversity of the locally adapted practices and crops grown.^[^ ^18^, ^34^, ^35^ ^]^ Shifting cultivation is important not just for economic reasons but also for cultural reasons where preservation of culture is more important than other concerns of environmentalists and governments.^[^ ^43^ ^]^ Evidently, under appreciation of the diversity of shifting cultivation practices and their linkage to indigenous ways of life have led to counterproductive policies in the past. The dependence on results from narrowly focused studies as argued by Mertz and co‐workers^[^ ^44^ ^]^ and recycling of stereotypical statements about shifting cultivation has also contributed to such policies. But, there are reports of successful practice where indigenous farmers are not under pressure from restrictive policies. For example, projects like the Nagaland Environment Protection for Economic Development that aimed to explicitly improve shifting cultivation instead of replacing it completely.^[^ ^24^ ^]^ Such a novel project can provide a better perspective and solutions to tackle indigenous population–forests conflicts.^[^ ^45^ ^]^ Our recent work in Northeast India^[^ ^34^, ^35^ ^]^ has revealed that shifting cultivation evolved as a part of the culture of indigenous peoples of the region, and the agricultural practices (Figure 2) are closely linked with their sociocultural practices and their beliefs. Similarly, empirical studies in Africa show that shifting cultivation in some areas have a lower impact and a smaller footprint than conventional practices.^[^ ^46^ ^]^ We believe that the abandonment of traditional ways of life of indigenous peoples due to the misconceptions around shifting cultivation would be a loss for the larger society.

## 4 Conclusion and Recommendations

After a careful review of the literature, we conclude that shifting cultivation still remains a complex and misunderstood form of land use. We also conclude that the dichotomy of opinions regarding the merits and demerits of shifting cultivation is the consequence of the attribution problem. We argue that shifting cultivation should be accepted as a rational land use system rather than labelling it as the “tropical deforestation culprit.” We also recommend a more nuanced understanding of local context and caution about generalizations on shifting cultivation and its linkage to deforestation. Effective governance, including customary institutions and co‐management involving indigenous peoples and local communities, can be an effective way to safeguard nature and its contributions to people.

## Conflict of Interest

The authors declare no conflict of interest.
